# Remission of Collagen-Induced Arthritis through Combination Therapy of Microfracture and Transplantation of Thermogel-Encapsulated Bone Marrow Mesenchymal Stem Cells

**DOI:** 10.1371/journal.pone.0120596

**Published:** 2015-03-16

**Authors:** He Liu, Jianxun Ding, Jincheng Wang, Yinan Wang, Modi Yang, Yanbo Zhang, Fei Chang, Xuesi Chen

**Affiliations:** 1 Department of Orthopedics, The Second Hospital of Jilin University, Changchun, P. R. China; 2 Key Laboratory of Polymer Ecomaterials, Changchun Institute of Applied Chemistry, Chinese Academy of Sciences, Changchun, P. R. China; 3 Academy of Translational Medicine, The First Bethune Hospital of Jilin University, Changchun, P. R. China; Georgia Regents University, UNITED STATES

## Abstract

The persistent inflammation of rheumatoid arthritis (RA) always leads to partial synovial hyperplasia and the destruction of articular cartilage. Bone marrow mesenchymal stem cells (BMMSCs) have been proven to possess immunosuppressive effects, and widely explored in the treatment of autoimmune diseases. However, poor inhibitory effect on local inflammatory state and limited capacity of preventing destruction of articular cartilage by systemic BMMSCs transplantation were observed. Herein, toward the classical type II collagen-induced arthritis in rats, the combination treatment of microfracture and *in situ* transplantation of thermogel-encapsulated BMMSCs was verified to obviously down-regulate the ratio of CD4^+^ to CD8^+^ T lymphocytes in peripheral blood. In addition, it resulted in the decreased levels of inflammatory cytokines, such as interleukin-1β, tumor necrosis factor-α and anti-collagen type II antibody, in the serum. Simultaneously, the combination therapy also could inhibit the proliferation of antigen specific lymphocytes and local joint inflammatory condition, and prevent the articular cartilage damage. The results indicated that the treatment programs could effectively stimulate the endogenous and exogenous BMMSCs to exhibit the immunosuppression and cartilage protection capability. This study provided a new therapeutic strategy for autoimmune inflammatory diseases, such as RA.

## Introduction

Rheumatoid Arthritis (RA) is an autoimmune disease, which mainly behaves as symmetry peripheral joint chronic inflammation, joint swelling and pain, articular cartilage damage and even deformity, and eventually leads to dysfunction [1]. The current therapeutic procedures, that is, mainly through oral administration of various drugs, focus on controlling inflammation and/or delaying or even preventing the progression of disease [2]. The symptoms of RA patients can be ameliorated partly, while the therapeutic effect on individuals still remains undesirable [3]. Therefore, so far, there is no effective disease-targeting treatment for RA patients, who have to bear sustaining pain and a risk of cartilage damage, and have the final fate of arthroplasty [4].

Mesenchymal stem cells (MSCs) are widely distributed in various tissues, including bone marrow, fat, synovial membrane, perichondrium, periosteum, *et al*. [5]. Among them, bone marrow mesenchymal stem cells (BMMSCs) have attracted extensive attention for their fascinating ability of differentiation into sundry cells, such as osteoblasts, chondrocytes and adipocytes, which have been applied in diversified tissue engineering [6]. As a typical example, our previous study confirmed that the full-thickness cartilage defect could be effectively repaired through the intra-articular admission of uncultured BMMSCs [7], and the local cell therapy for cartilage defects of RA seem to be a reasonable scheme. What must be considered is that RA patients are confronted with the risk of cartilage re-injury. In addition, synovial fluid obtained from RA patients shows significantly reduced BMMSCs recruitment compared to normal donors, which is an important origin of cartilage restoration [8]. Therefore, both inhibiting local joint inflammation and preventing cartilage destruction synchronously are rather tough.

Interestingly, BMMSCs also exhibit powerful function on regulating immune response, including suppressing the proliferation of T lymphocytes and inhibiting inflammatory mediators [9]. These properties give BMMSCs the opportunity to restrict various autoimmune diseases, such as RA, multiple sclerosis, systemic lupus erythematosus, crohn's disease, and so on [10–12]. Autologous BMMSCs transplantation sounds perfect for the treatment of RA, while the capability of self-renew and differentiation of BMMSCs obtained from RA patients significantly reduced compared with that from healthy people [13], which are also affected by age [14]. It indicates that the additional BMMSCs are demanded to remedy the flaws in RA. Fortunately, BMMSCs lack the expression of major histocompatibility complex class II and immune stimulating molecules, even under stimulation are still nonimmunogenic, so BMMSCs can escape immune surveillance and won't cause graft rejection [15].

To date, in consideration of that RA is a systemic disease, the approaches that using allogeneic BMMSCs grafting for systemic treatment on RA were mainly performed *via* intravenous or intraperitoneal injection [16–19], while the localized administration is rarely attempted [20]. Once the BMMSCs are transplanted into articular cavity, the cells mainly obtain the nutrition and oxygen from the synovial fluid [21], and the inflammatory microenvironment will certainly hamper the cell viability. To address above problem, microfracture through the non-weight-bearing area is created. In practice, the penetration from subchondral bone marrow causes the formation of blood clot, which allows endogenous BMMSCs, various cytokines, oxygen and nutrients to access the channel [22], and the implanted BMMSCs can enter bone marrow to perform immunosuppressive function meanwhile.

**Fig 1:**
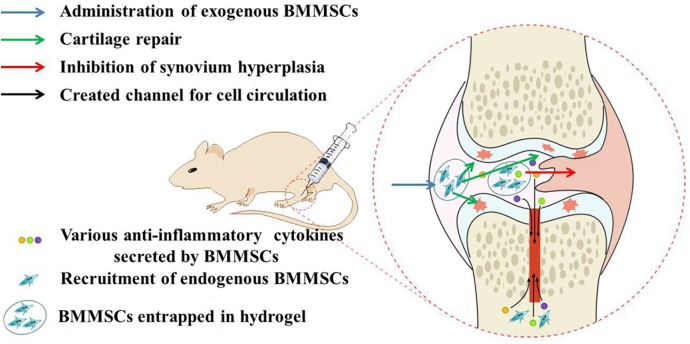
Schematic illustration of possible mechanisms for remission of collagen-induced arthritis.

Three-dimensional scaffold can provide a relatively stable space for the expansion of BMMSCs, and thermosensitive hydrogels are one of the vastly applied bio-scaffold in tissue engineering, including cartilage repair, bone regeneration and cardiac restoration [23–26]. Among them, the thermogel based on poly(D,L-lactide-*co*-glycolide)-*block*-poly(ethylene glycol)-*block*-poly(D,L-lactide-*co*-glycolide) triblock copolymers (PLGA-*b*-PEG-*b*-PLGA) has shown outstanding potential for clinical applications due to their excellent tunable gelation behavior, biocompatibility and biodegradability [27,28]. In addition, our previous work confirmed that the PLGA-*b*-PEG-*b*-PLGA thermogel supports excellent adhesion and proliferation of BMMSCs [29]. In this work, the influence of intra-articular transplantation of exogenous BMMSCs entrapped in PLGA-*b*-PEG-*b*-PLGA thermogel accompanying with endogenous MSCs from bone marrow after microfracture on systemic immunosuppression and protection of surrounding cartilage under arthritic condition was clarified in rat model of collagen-induced arthritis (CIA), described as Fig. 1. It aimed to explore a novel procedure to make up for the deficiency of current clinical treatments.

## Materials and Methods

### Reagents

PLGA-*b*-PEG-*b*-PLGA triblock copolymer (number-average molecular weight (*M*_n, NMR_) = 4200 g/mol, LA/GA = 75:25, mol/mol) was synthesized as our previous works [30]. Percoll density gradient, 3-(4,5-Dimethyl-thiazol-2-yl)-2,5-diphenyl tetrazolium bromide (MTT) and interleukin-1β (IL-1β), tumor necrosis factor-α (TNF-α) and anti-collagen type II antibody (anti-COL-II Ab) enzyme-linked immunosorbent assays (ELISAs) kit were purchased from Sigma-Aldrich, USA. Chick type II collagen (COL-II) and complete Freund's adjuvant (CFA) were acquired from Chondrex, USA. FITC-conjugated anti-CD3, PE-conjugated anti-CD4, and APC-conjugated anti-CD8 mAbs were obtained from eBioscience, San Diego, CA. Dimethyl sulfoxide (DMSO) was stored over calcium hydride (CaH_2_) and purified by vacuum distillation with CaH_2_.

### Animal Models of CIA

**Fig 2:**
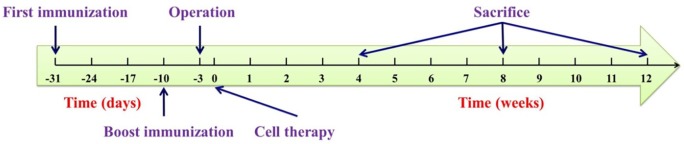
Schematic chronogram of experimental design.

As shown in Fig. 2, the protocol was approved by the Animal Care and Use Committee of Second Hospital, Jilin University, and all efforts were made to minimize suffering. 36 male Sprague-Dawley (SD) rats (200 ± 20 g, 6 weeks) were supplied by the Experimental Animal Center of Jilin University. After adaptive feeding for a week, 36 SD rats were divided into 4 groups referred to as CON, BLA, GEL and BMC. Detailed information was described in Table 1. All rats received a subcutaneous injection of the emulsion prepared by mixing 100.0 μg of COL-II in 0.1 N acetic acid with 50.0 μg of CFA at the base of tail. An additional immunization boost was given at day 21 by half the dosage of emulsion at the base of tail.

**Table 1:** The procedures of different experimental groups.

Study group	Operation and Implanted contents	Number of rats and sacrificed time (weeks post transplantation)
CON	No operation	3(4w) + 3(8w) + 3(12w)
BLA	Operation and PBS	3(4w) + 3(8w) + 3(12w)
GEL	Operation and gel	3(4w) + 3(8w) + 3(12w)
BMC	Operation and BMMSCs	3(4w) + 3(8w) + 3(12w)

### Surgical Procedure

SD rats of BLA, GEL and BMC groups (*n* = 27) underwent operations at one week post boost injection. In brief, rat was anaesthetized with 3% (w/v) pentobarbital sodium (30.0 mg/kg) and performed a longitudinal cut *via* a medial approach of left knee. The patella was dislocated and the joint was flexed to exposure the tibial plateau. And then, a hole (diameter = 1 mm) was drilled in the center of tibial plateau until the articular cavity was extended to subchondral bone marrow. Finally, the patella was reset and the wound was closed in layers. The rats of CON group underwent sham operation without drilling. Animals were allowed to move freely after surgery. The rats received gentamicin by intramuscular injection (1.5 mg/kg) each day for 3 times post-operatively, and no rats were found with skin infection or dead during the experiment.

### Extraction and Culture of BMMSCs

Bone marrow was harvested from the tibia and femur of a male immature SD rat (50 g, 3 weeks). The mononuclear cells were isolated by percoll density gradient, and plated on culture dish in low-glucose Dulbecco's modified Eagle's medium (LG-DMEM) supplemented with 10% (v/v) fetal bovine serum (FBS) and 1% (w/v) penicillin-streptomycin at 37°C. Non-adherent cells were removed 4 days later, and the attached BMMSCs were harvested. When cells grew to ∼ 80% saturation, the adherent cells were digested with 0.25% trypsin/EDTA at 37°C for 3 min and passaged, and the 3^rd^ passage of BMMSCs were readied for use.

### Administration of BMMSCs Loaded in PLGA-*b*-PEG-*b*-PLGA Thermogel

Cell therapy with allogeneic BMMSCs was performed 3 days after the surgery. Rats were treated with the suspension of 5 × 10^6^ allogeneic BMMSCs mixed in 100.0 μl of PLGA-*b*-PEG-*b*-PLGA copolymer solution in phosphate-buffered saline (PBS) at 4°C (20 wt.%; noted as BMC, *n* = 9) or treated with 100.0 μl of PLGA-*b*-PEG-*b*-PLGA thermogel alone (noted as GEL, *n* = 9). The group treated with 100.0 μl of PBS was named as blank (BLA, *n* = 9), and the group without surgery treated with 100.0 μl of PBS was named as control (CON, *n* = 9). The groups were described in detail in Table 1. Rats were monitored for the signs of arthritis onset, and the disease scores of CIA were assessed weekly using the previously described procedures on a scale of 0 − 3 based on the level of erythema, swelling or joint rigidity, where 0 = no swelling, 1 = slight swelling and erythema, 2 = pronounced edema, and 3 = joint rigidity. Each limb of a rat was scored separately, the total score of each rat was calculated and presented as the average value by group (Arthritis Index) [31].

### COL-II-Specific Proliferation Lymphocytes Derived from Draining Lymph Node (DLN) and Spleen

The rats were sacrificed at 4, 8 or 12 weeks post-transplantation. Lymphocytes from DLN and spleen were isolated at the time of sacrifice, and cells were supplemented and cultured as previously described to measure specific cell proliferation [32]. In brief, each culture was performed for 2 × 10^5^ lymphocytes/well in 96-well plate, and 200.0 μl of complete LG-DMEM was added in triplicate. CD11c+ dendritic cells were separated from spleen of the same rat, and were pre-treated with mitomycin C to prohibit their own proliferation, then added as antigen-presenting cells with equal number of lymphocytes with the stimulation of COL-II at a concentration of 5.0 μmol or otherwise. The lymphocytes cultured only with non-T cells served as negative control. The plates were incubated in a humidified atmosphere of 5% CO_2_ at 37°C for 72 h, and then 20.0 μl of MTT was added to each well to co-culture for 4 h. The supernatant was removed, and 150.0 μl of dimethyl sulfoxide was added. The absorbance of above medium was measured at 490 nm on a Bio-Rad 680 microplate reader (Model 550, Hercules, CA, USA).

### Flow Cytometry Analysis of T Lymphocyte Subsets

1.0 ml of peripheral blood was collected from the tail vein of SD rat with an Eppendorf tube containing 50.0 μl of heparin sodium solution (1000.0 IU/ml) at the time point of sacrifice (*i*.*e*., week 4, 8 and 12). Flow cytometry analysis was performed to detect T lymphocyte subsets in peripheral blood using FITC-conjugated anti-CD3, PE-conjugated anti-CD4 and APC-conjugated anti-CD8 mAbs. Cells were analyzed on a FACSCalibur flow cytometer (BD Biosciences, USA), and the data were collected for 10,000 events per sample with CellQuest Pro software (BD Biosciences, USA).

### Measurement of Serum Cytokines and Specific Anti-Collagen Antibody

Peripheral blood was configured to collect the upper serum for the detection of cytokines, IL-1β, TNF-α and COL-II Ab by ELISAs following the instructions of ELISA assay kits. The concentration of each protein was calculated from the standard curve.

### Macroscopic and Histological Assessment

At 4, 8 and 12 weeks post-transplantation, the rats were sacrificed, and both the distal femurs and surrounding synovium were isolated. The femurs were carefully examined and photographed. After gross examination, distal femurs were collected and fixed in 4% (w/v) paraformaldehyde, and decalcified and embedded for paraffin-sectioning (5 μm). Hematoxylin and eosin (H&E) staining was performed to evaluate the situation of surrounding synovial membrane and cartilage. The morphological features of the synovium were assessed in H&E-stained slices according to the criteria described in Table 2 [33]. Three sections from each sample were randomly chosen and scored by two blinded observers, and the possible maximum score is 9.0. As depicted in Table 3, the modified ORASI score system was applied to evaluate the cartilage status microscopically [34]. In addition, the thickness of hyaline cartilage (HC, zone from cartilage surface to tidemark) and calcified cartilage (CC, zone from tidemark to surface of subchondral bone plate) were measured with imagine analysis software of Nano Measurer 1.2, and the ratios of thickness of HC to CC were also calculated.

**Table 2:** Morphological features of synovium.

Feature	Score
A. Hyperplasia or enlargement of synovial lining cell layer	
1. Absent	score = 0
2. Slight enlargement (two to three cell layers). Giant cells are very rare	score = 1
3. Moderate enlargement (four to five cell layers). Some giant cells or lymphocytes	score = 2
4. Strong enlargement (more than six cell layers). Giant cells and lymphocytes are frequent	score = 3
B. Inflammatory infiltration	
1. Absent	score = 0
2. Slight inflammatory infiltration (diffusely located single cells and small perivascular aggregates of lymphocytes and/or plasma cells)	score = 1
3. Moderate inflammatory infiltration (perivascular and/or superficial lymphatic aggregates, and small sized lymphatic follicles without germinal center may be observed)	score = 2
4. Strong inflammatory infiltration (lymphatic follicles with germinal center and/or confluent subsynovial lymphatic infiltration)	score = 3
C. Activation of synovial stroma/pannus formation	
1. Absent	score = 0
2. Slight synovial stroma activation (low cellularity with slight edema, slight fibrosis with some fibroblast, no giant cells)	score = 1
3. Moderate synovial stroma activation (moderate cellularity with a moderate density of fibroblasts, endothelial cells, and giant cells may be detected)	score = 2
4. Strong synovial stroma activation (high cellularity with dense distribution of fibroblasts and endothelial cells, and giant cells are abundant)	score = 3

**Table 3:** Modified OARSI scores to evaluate the cartilage status microscopically.

Feature	Score
A. Structure	
0. Normal	score = 0
1. Slight surface irregularities	score = 1
2. Moderate surface irregularities	score = 2
3. Severe surface irregularities	score = 3
4. Clefts/fissures into transitional zone (one-third depth)	score = 4
5. Clefts/fissures into radial zone (two-thirds depth)	score = 5
6. Clefts/fissures into calcified zone (full depth)	score = 6
7. Fibrillation and/or erosion to transitional zone (one-third depth)	score = 7
8. Fibrillation and/or erosion to radial zone (two-thirds depth)	score = 8
9. Fibrillation and/or erosion to calcified zone (full depth)	score = 9
10. Fibrillation and/or erosion to subchondral bone	score = 10
B. Cellularity	
0. Normal	score = 0
1. Increase or slight decrease	score = 1
2. Moderate decrease	score = 2
3. Severe decrease	score = 3
4. No cells present	score = 4
C. Chondrocyte cloning	
0. Normal	score = 0
1. Several doublets	score = 1
2. Many doublets	score = 2
3. Doublets and triplets	score = 3
4. Multiple cell nests	score = 4

### Statistical Analysis

All experiments were performed at least three times, and the data were expressed as means ± standard deviation (SD). The statistical analysis was performed with the software SPSS 13.0 (SPSS Inc., Chicago, IL, USA). *p* < 0.05 was considered statistically significant.

## Results

### Prevention of Severe Tissue Damage in CIA Models by BMMSCs

**Fig 3:**
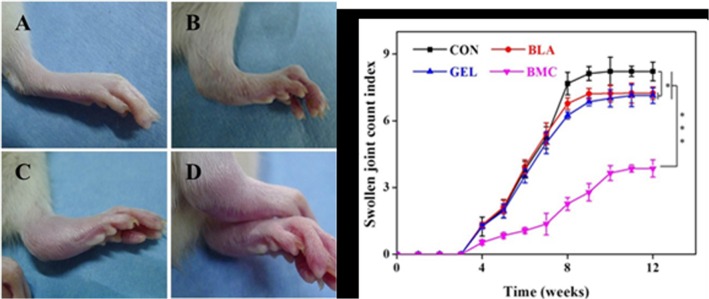
Representative photos of different degrees of CIA severity (A − D); disease scores in individual male SD rats after immunization with type II collagen (COL-II) and treatment with allogeneic BMMSCs (E). Each limb of a rat was scored separately, and the average score for each animal was calculated.

In this work, 36 SD rats were immunized with COL-II to generate CIA models. One week after boost immunization, 27 SD rats were randomly selected to perform the operation and divided into 3 groups (BMC, GEL, BLA), the remaining 9 SD rats were noted as CON group. Then the BMC group accepted the administration of 5 × 10^6^ allogeneic BMMSCs mixed in 100.0 μl of PLGA-*b*-PEG-*b*-PLGA copolymer solution, and the GEL group accepted equal volume of thermogel alone, the group treated with 100.0 μl of PBS was named as BLA. The signs of CIA with different degrees (Fig. 3A − D) were observed as time goes on, and the mean disease score of all CIA models was 6.6 at 12 weeks post cell therapy.

After the combination of microfracture and transplantation of thermogel-encapsulated bone marrow mesenchymal stem cells, the BMC group showed significantly mildest symptoms of RA with disease score at 3.9 ± 0.4 compared with all the other groups (*i*.*e*., CON, BLA and GEL). In addition, both the BLA (7.2 ± 0.3) and GEL (7.1 ± 0.4) groups exhibited relatively slighter symptom of swollen joint in contrast with CON group (8.2 ± 0.4) (Fig. 3E).

### BMMSCs-Induced Hyporesponsiveness of Lymphocytes

**Fig 4:**
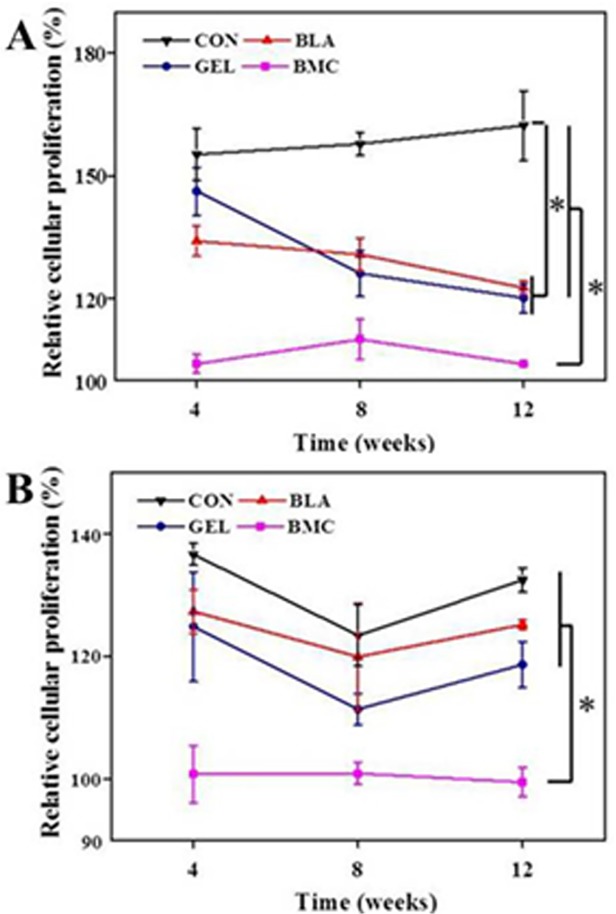
Relative proliferation of lymphocytes isolated from DLN (A) and spleen (B) of CIA rats stimulated by 5.0 μmol of COL-II after treatment with BMMSCs. Data are represented as means ± SD (*n* = 3).

The proliferation experiment of lymphocytes revealed that the treatment with allogeneic BMMSCs induced the obvious hyporesponsiveness of lymphocytes from DLN (Fig. 4A) and spleen (Fig. 4B) of immunized rats at 4, 8 or 12 weeks, whereas the lymphocytes from other groups proliferated markedly when stimulated with COL-II under the same conditions (*p* < 0.05). In addition, the rats in BLA and GEL groups also exhibited remarkable immunosuppressive characters in DLN experiment (*p* < 0.05), but it was less obvious toward splenocytes.

### Suppression of CD4^+^ T Lymphocytes Detected by Flow Cytometry

**Fig 5:**
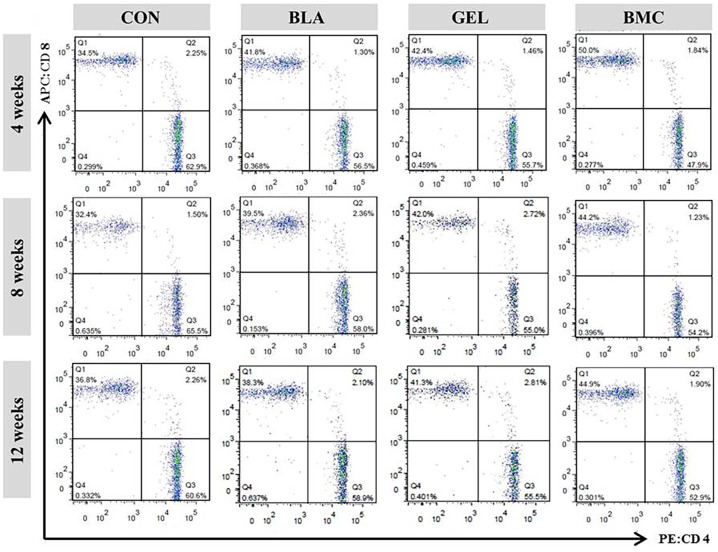
Flow cytometry analysis of CD3^+^ T lymphocyte subsets in peripheral blood analyzed using FITC-conjugated anti-CD3, PE-conjugated anti-CD4, and APC-conjugated anti-CD8 mAbs.

The subsets of T lymphocytes including CD3^+^, CD4^+^ (inducer/helper) and CD3^+^, CD8^+^ (cytotoxic/suppressor) in peripheral blood were measured by flow cytometry (Fig. 5). The result revealed that the ratio of CD4^+^ to CD8^+^ lymphocytes, reflecting the balance of immunoregulatory cells, in BMC group was lower than that in other groups (*p* < 0.05). In addition, the BLA and GEL groups exhibited relatively less disparity compared with CON group. A upward trend of the ratio of CD4^+^ to CD8^+^ in BMC group was observed as time goes on, indicating fluctuant therapeutic effect after the combination treatment and a slow progression of CIA after 4 weeks post-treatment.

### Down-Regulated Expression of Inflammatory Cytokines Following Treatment with BMMSCs

**Fig 6:**
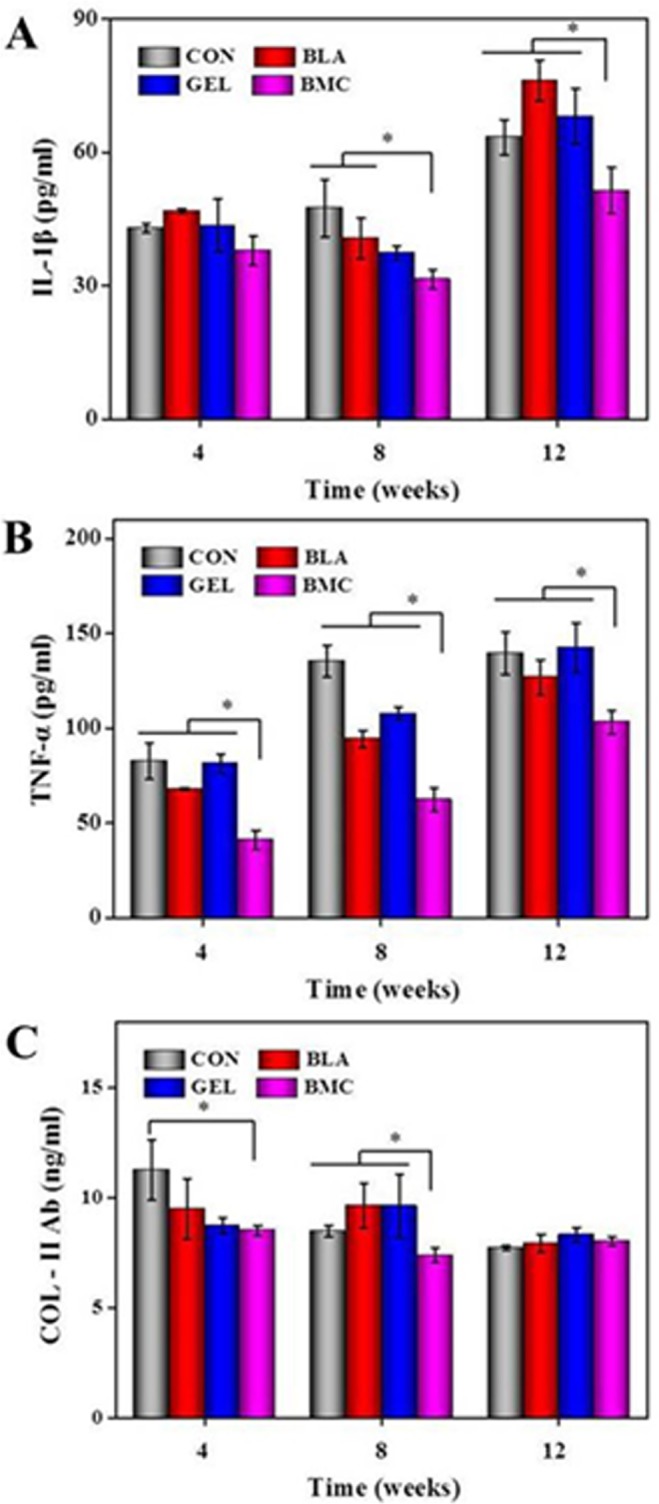
Concentrations of IL-1β, TNF-α and anti-COL-II Ab in serum analyzed by ELISA. Data are represented as means ± SD (*n* = 3).

The mechanisms underlying the decrease in severity of CIA following operation and the transplantation of BMMSCs were deliberated, and the effect on the production of inflammatory factors that are closely linked to synovium inflammation was also evaluated. The results showed that the transplantation of BMMSCs significantly reduced the expression of inflammatory cytokines, such as IL-1β and TNF-α (Fig. 6A and 6B). As previously reported, the antibody against collagen-rich synovium is involved in the pathogenesis of CIA [35]. In this work, the administration of BMMSCs resulted in the reduced serum levels of COL-II Ab (Fig. 6C), suggesting that the local anti-inflammatory effect of administrated BMMSCs was accompanied with the down-regulation of systemic inflammatory response.

### Reduction of Synovial Hyperplasia and Cartilage Damage

#### Macroscopic appearance of joint

**Fig 7:**
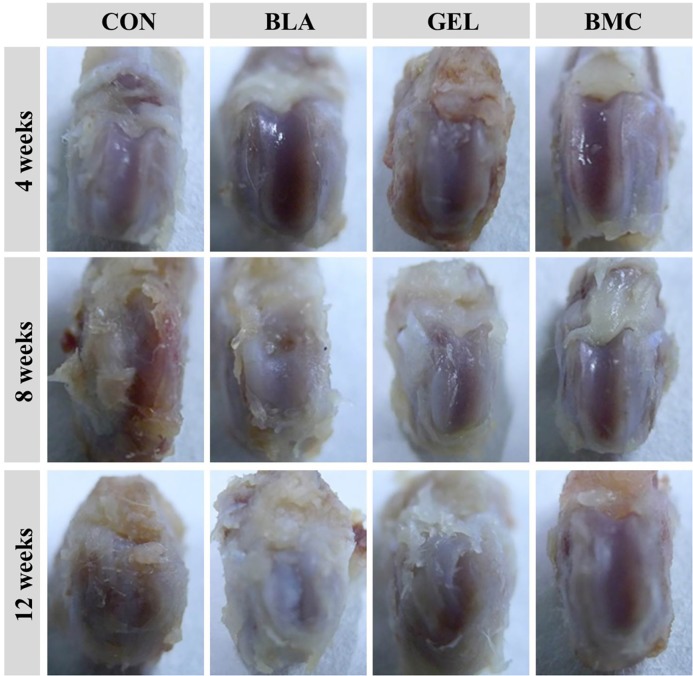
Typical appearance of articular cartilage at different time points after implantation.

The macroscopic appearance of joint can reflect the degree of cartilage destruction intuitively. In this study, the results demonstrated that there was no cartilage lesion at 4 weeks in any groups. However, the gross findings in the control group displayed the broad areas of cartilage damage with irregular surface fibrillation at 8 weeks, which was also found in BLA and GEL groups at 12 weeks. The BMC group demonstrated the best outcomes and showed generally intact surfaces, even if slight surface fibrillation was revealed at 12 weeks (Fig. 7).

#### Histological analysis of synovium and cartilage

**Fig 8:**
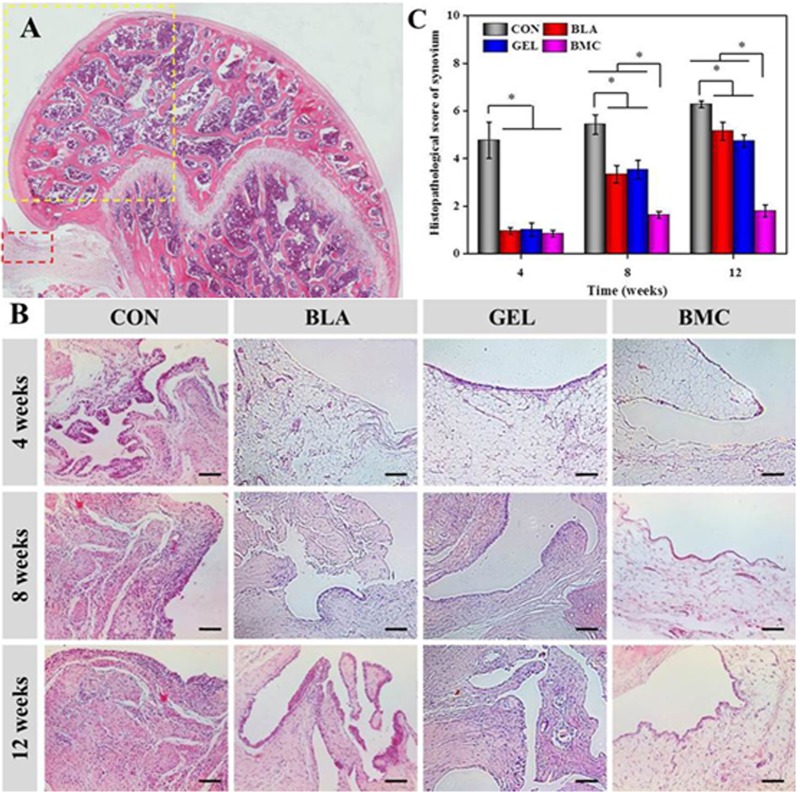
Longitudinal section of distal femur indicating the region of interest (black box and yellow box for synovium and cartilage, respectively) (A); synovium was stained with H&E (scar bar = 100 μm) (B), and the histopathological score was evaluated at 4, 8 and 12 weeks post-transplantation (C).

No inflammatory cells infiltrated the fibrous and adipose tissues underlying the synovial lining of the synovial fringes, and minimal hyperplasia of the synovial lining were observed in the BMC group. The histopathological scores of synovium were 0.8 ± 0.1, 1.6 ± 0.1 and 1.8 ± 0.3 at 4, 8 and 12 weeks, respectively. The result indicated the absence of inflammatory reaction and slight synovial hyperplasia. However, the hyperplasia of synovial lining and inflammatory cells suffusing in synovium in CON group with histopathological scores of 4.8 ± 0.8, 5.4 ± 0.4 and 6.3 ± 0.2 at 4, 8 and 12 weeks, respectively, were observed throughout the detection stage. At the same time, the same symptoms were observed at the later period of BLA with histopathological scores of 3.3 ± 0.4 and 5.1 ± 0.4 at 8 and 12 weeks, respectively, and GEL groups with those of 3.5 ± 0.4 and 5.7 ± 0.3 at 8 and 12 weeks, respectively (Fig. 8).

**Fig 9:**
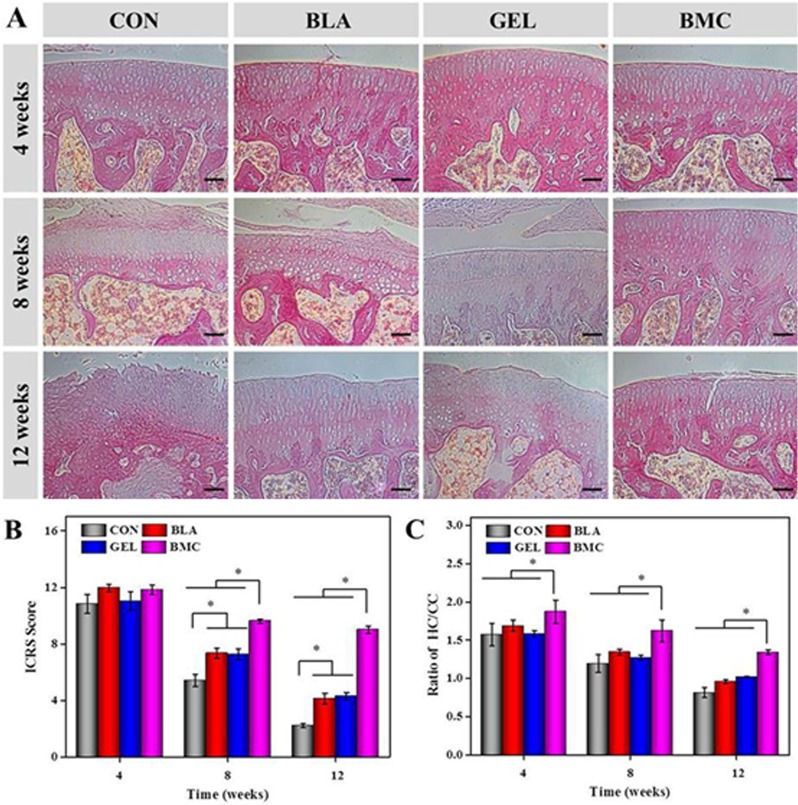
H&E staining of cartilage sections (scar bar = 100 μm) (A), the modified OARSI score of microscopic observation (B) and the ratio of the thickness of hyaline cartilage to calcified cartilage were evaluated at 4, 8 and 12 weeks post-transplantation (C).

The cartilage histological results in CON group revealed the progression of CIA up to the deep zones of the cartilage layers with high OARSI score. The results in both BLA and GEL groups also demonstrated the slower progression of CIA changes with minor cartilage destruction. The sample in BMC group exhibited no surface fibrillation and minimal degree of cartilage lesion (Fig. 9A and 9B). The thinning of cartilage during RA progression could occur from both directions by both surface degradation and the increase in cartilage calcification or hypertrophy from the cartilage/bone interphase [36]. The thickness of calcified cartilage in these rats of CON, BLA and GEL groups with the tidemark moving closer to the articular surface were greater than that in the rats of BMC group (Fig. 9C). In short, these results indicate that the implantation of BMMSCs loaded in PLGA-*b*-PEG-*b*-PLGA thermogel exhibits excellent anti-inflammatory and cartilage protective effect.

## Discussion

As above mentioned, RA is a systemic disease. If it is not treated properly, RA can eventually lead to the irreversible severe articular cartilage damage [1]. The patients who want to get rid of unbearable pain need joint plasty or joint fusion, and one of the most important remedial measures is to prevent the cartilage destruction [20]. MSCs have attracted more and more attention due to the excellent function of cartilage repair and immune-regulatory. Intra-articular transplantation is the most immediate treatment approach for arthritis and other joint disorders. However, the localized cell therapy can avoid some problems, such as unnecessary organs involvement and tumorigenic issues [37], and can also reduce the total amount of cells used in the treatment. Recently, it has been proven that the full-thickness defect extending into the marrow cavity can recruit intrinsic BMMSCs to compensate for a deficiency in the number or function of MSCs that occur in the injured tissues [8]. In addition, the stimulation of marrow is based on the provocation of inflammation, which leads to the mobilization of endogenous bone marrow-derived MSCs [38]. In this work, a new way to prevent progressive RA from severe cartilage damage was attempted. Firstly, a channel linking bone marrow to articular cavity was created to enhance the circulative capacity of intrinsic BMMSCs, and then the exogenous BMMSCs encapsulated in PLGA-*b*-PEG-*b*-PLGA thermogel were implanted. Under the stimulation of arthritic condition, the mobilized BMMSCs can secrete various paracrine factors to regulate the immune response locally or through hematologic system and node pathways to exert their immune regulatory function systemically.

Joint swelling is a common symptom for RA patients, which is mainly caused by the synovial hyperplasia and joint effusion [39]. In this study, the count indexes of swollen joint revealed that the vast majority of immunized rats developed irreversible bone or cartilage erosions except for BMC group, whose degree of disease score was lower than those of non-treatment groups, while higher than normal rats. It indicated that the treatment of BMMSCs could inhibit, but could not completely eliminate synovial hyperplasia, which had been confirmed by the histopathological analysis that revealed the reversible symptom of synovial inflammation. The operation, which leads to the convenient situation for the recruitment of endogenous BMMSCs, can ameliorate the condition of CIA in some extent.

In order to investigate the pathway of local BMMSCs therapy, the specific stimulation of lymphocytes and splenocytes by COL-II was assessed. The outcomes proved that BMMSCs behaved their immunosuppressive function through lymph metastasis or blood circulation with an obvious declining inhibitory effect. The flow cytometry analysis ingenuity demonstrated that the transplantation of BMMSCs can influence the systemic situation of inflammatory response, and the ratio of CD4^+^ to CD8^+^ T lymphocytes significantly decreased during the experiment performance in BMC group compared with that of other groups. Whereas, the ratios of CD4^+^ to CD8^+^ T lymphocytes in BLA and GEL groups were lower than that in CON group, which may account for the intervention of intrinsic BMMSCs.

The proinflammatory cytokines, such as IL-1β, may affect the chondrogenic and anabolic ability of cartilage, and are considered to display catabolic effects and stimulate proteinases. Subsequently, they may result in extracellular cartilage matrix degradation that impedes cartilage repair, maturation and lateral integration [40]. TNF-α stimulates the proliferation of synovial cells, and induces the release of other pro-inflammatory cytokines, leading to joint destruction [41]. Anti-COL-II Ab, which is secreted by activated B lymphocytes under the stimulation of COL-II, is a key index indicating the severity of collagen induced arthritis [42]. The decreased levels of IL-1β, TNF-α and anti-COL-II Ab in serum suggested that the transplantation of BMMSCs significantly attenuated the inflammatory situation through the down-regulation of pro-inflammatory cytokines and antigen specific antibody.

As we all know, synovial fibroblasts (SFs) are one of the most abundant resident cells in synovium. In the synovium of RA patients, the abnormal proliferation of SFs occurs by either acquired stimulation or the recruitment of variable ancestral cells, and amount of evidences support that expansion of SFs contributed to the pathogenesis of RA [43]. SFs can respond to a variety of cytokines, particularly TNF-α, and SFs can secrete various inflammatory mediators that may result in cartilage destruction [44,45]. The cytokine-induced cascade responses lead to the up-regulation of the synthesis of cytokines, pro-angiogenic factors, chemokines and factors associated to enhanced invasiveness and cartilage destruction [46]. Therefore, there is a closely connection between cytokines, synovium and cartilage. A major objective of this paper is to check the efficacy of MSCs therapy on cartilage protection, and the basic performance of cartilage destruction can be examined by the direct observation of joint surface and histopathological staining. It turned out that the synovial hyperplasia was observed at 4 weeks in CON group, and 8 weeks in BLA and GEL groups, simultaneously, the cartilage destruction was observed at 8 weeks in CON group, and 12 weeks in BLA and GEL groups. Fortunately, there was no synovial hyperplasia or cartilage destruction in BMC group over the entire detection period. All the results were future confirmed by the H&E staining of surrounding synovium and cartilage. Apart from the synovial hyperplasia, the infiltration of inflammatory cells was found in the deep layer of synovium of CON, BLA or GEL group except for BMC group. Those results indicated that the intrinsic BMMSCs can delay the progress of synovial hyperplasia and cartilage destruction, while the transplantation of BMMSCs in thermogel can inhibit synovial hyperplasia and the infiltration of inflammatory cells and thereby ameliorate the cartilage integrity synchronously.

There may be two possible mechanisms to explain the above declaration. Firstly, a previous study reported that MSC-differentiated chondrocytes owned the same immunological characters as the undifferentiated MSCs. It suggests that MSC-chondrocytes might suppress inflammatory factors and prevent re-destruction while exerting their repairing effects once being implanted into RA joints [47]. Another study revealed that the differentiated MSCs, such as fibroblasts, could inhibit T cell proliferation *in vitro*, while showed no immunosuppressive effect *in vivo* [48]. Actually, MSCs responding to the irritation of inflammation can be influenced by the presence of inflammatory factors. The addition of TNF-α can reverse the immunosuppressive effect of MSCs on T cells proliferation. It suggests that the interactions between MSCs and arthritic microenvironment are reciprocal [49]. Beyond that, the channel between articular cavity and subchondral bone marrow is blocked by regenerative tissue, which can certainly impede the interaction between articular cavity and bone marrow. It may explain why the immunosuppressive effect of BMMSCs cannot persist for a long period. In previous studies, the intrinsic MSCs were rarely investigated rather than being utilized. Despite the less remarkable effects than those of exogenous BMMSCs admission, to a certain extent, the recruitment of endogenic BMMSCs alleviate the RA-linked symptoms, down-regulate the hyporesponsiveness of lymphocytes and slow the progress of RA, which all are verified by the histological examination. Nevertheless, the mechanisms remain further investigation.

Further research on the BMMSCs-located microenvironment and regulatory mechanism will be of central importance. The advanced studies should focus on how to take advantage of BMMSCs accompanied with the endogenous progenitor cells to modulate the local microenvironment for the purposes of protection or repair of articular cartilage. In addition, the MSCs from different sources, such as umbilical cord and adipose, also exhibit excellent therapeutic benefits on RA [50], and should be employed to optimize the above treatment program.

## Conclusions

In this study, a novel strategy by the combination of microfracture and local BMMSCs transplantation with a biodegradable PLGA-*b*-PEG-*b*-PLGA thermogel for the treatment of CIA were presented. Notably, the endogenous from microfracture and implanted exogenous BMMSCs can synergistically reduce the ratio of CD4^+^ to CD8^+^ T lymphocytes in peripheral blood and result in decreased levels of inflammatory cytokines. Moreover, the inhibitory effects on the proliferation of antigen specific lymphocytes and local articular inflammation, as well as the protective role on cartilage of the BMMSCs were revealed. While, the recruitment of endogenic BMMSCs through microfracture exerted lower effectiveness in these regards. Based on the above results, the MSCs transplantation combined with microfracture will present great hope in relieving the disease burden of RA patients.
